# Enhanced representation of natural sound sequences in the ventral auditory midbrain

**DOI:** 10.1007/s00429-020-02188-2

**Published:** 2020-12-14

**Authors:** Eugenia González-Palomares, Luciana López-Jury, Francisco García-Rosales, Julio C. Hechavarria

**Affiliations:** Institute for Cell Biology and Neuroscience, Goethe University, 60438 Frankfurt am Main, Germany

**Keywords:** Inferior colliculus, Auditory midbrain, Mutual information, Natural sounds, Brain-stimulus synchrony

## Abstract

The auditory midbrain (inferior colliculus, IC) plays an important role in sound processing, acting as hub for acoustic information extraction and for the implementation of fast audio-motor behaviors. IC neurons are topographically organized according to their sound frequency preference: dorsal IC regions encode low frequencies while ventral areas respond best to high frequencies, a type of sensory map defined as tonotopy. Tonotopic maps have been studied extensively using artificial stimuli (pure tones) but our knowledge of how these maps represent information about sequences of natural, spectro-temporally rich sounds is sparse. We studied this question by conducting simultaneous extracellular recordings across IC depths in awake bats (*Carollia perspicillata*) that listened to sequences of natural communication and echolocation sounds. The hypothesis was that information about these two types of sound streams is represented at different IC depths since they exhibit large differences in spectral composition, i.e., echolocation covers the high-frequency portion of the bat soundscape (> 45 kHz), while communication sounds are broadband and carry most power at low frequencies (20–25 kHz). Our results showed that mutual information between neuronal responses and acoustic stimuli, as well as response redundancy in pairs of neurons recorded simultaneously, increase exponentially with IC depth. The latter occurs regardless of the sound type presented to the bats (echolocation or communication). Taken together, our results indicate the existence of mutual information and redundancy maps at the midbrain level whose response cannot be predicted based on the frequency composition of natural sounds and classic neuronal tuning curves.

**Supplementary Information:**

The online version contains supplementary material available at 10.1007/s00429-020-02188-2.

## Introduction

Animals depend greatly on acoustic signals to interact with the environment and other life beings. Encoding acoustic information in the auditory system is a fundamental step leading to the production of behavioral responses in everyday scenarios (Ryan et al. 1985; Brudzynski 2013; Jiang et al. 2017; Liévin-Bazin et al. 2018). The latter could determine the animals’ well-being and their capacity to adapt to environmental pressures.

The main aim of this article is to study the representation of natural sounds in the auditory midbrain (inferior colliculus, IC). The IC is an important integration hub in the auditory pathway that has been linked to the production of fast audio-motor behaviors instrumental for animal survival (Covey et al. 1987; Casseday and Covey 1996; Malmierca 2004). This structure is also a target area for auditory prostheses that benefit deaf patients who cannot sufficiently profit from cochlear implants (Colletti et al. 2007; Lim et al., 2007, 2008). Albeit the IC has been studied extensively at the anatomical and functional levels (Casseday et al. 2002; Malmierca 2004; Simmons et al. 2020), our knowledge of how neurons within this structure represent natural sound streams is still sparse.

We relied on bats as experimental animal model to assess how natural sound sequences are represented simultaneously across IC depths. Bats represent an excellent animal model for auditory experiments because of their rich soundscape, which includes echolocation (sound-based navigation) and multiple types of communication sounds (Wilkinson and Boughma 1998; Schnitzler et al. 2003). The latter are used to maintain hierarchies in the colonies, to communicate with infants and to alert other individuals about potentially dangerous/uncomfortable situations (Balcombe and McCracken 1992; Gadziola et al. 2012; Knörnschild et al. 2013).

**Fig. 1:**
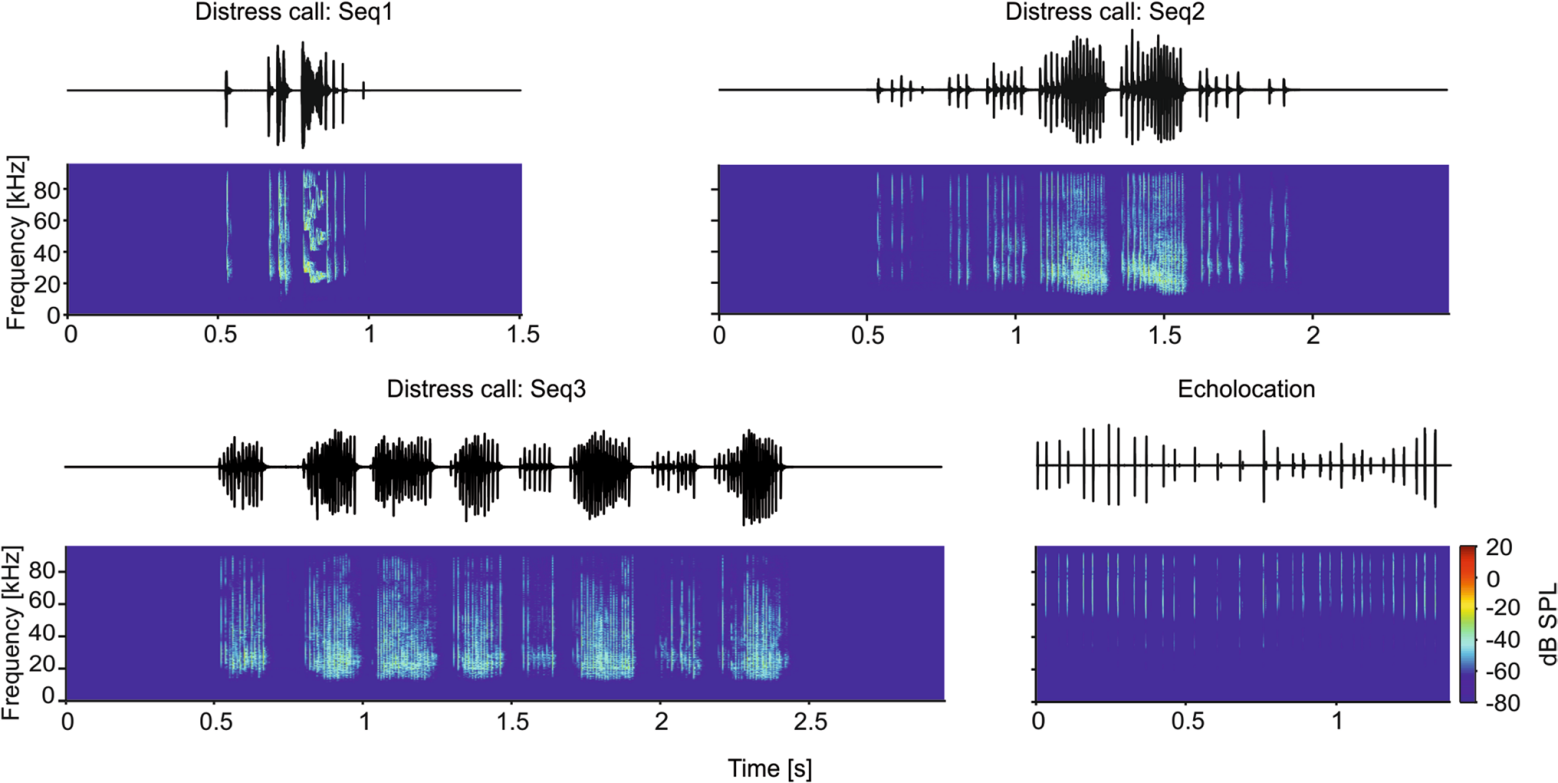
Oscillograms and spectrograms of the natural calls used as stimuli, three distress calls (Seq1, Seq2 and Seq3) and one biosonar call (echolocation)

The auditory system of bats has been heavily studied in the last decades but, at present, no consensus exists as to whether communication and echolocation sounds can be represented by the same neurons (Kössl et al. 2015). In the bat species *Carollia perspicillata* (the species of choice for this study), there is a clear dissociation in the frequency domain between communication and biosonar sounds used for orientation. The former cover the low-frequency portion of the bat soundscape, with the power of individual syllables peaking at frequencies close to 20 kHz, while the latter carry most energy in the high-frequency band between 45 and 100 kHz (Fig. 1; Brinkløv et al. 2011; Hechavarría et al. 2016a, b).

The bat IC follows the general mammalian plan with a dorsolateral–ventromedial tonotopic arrangement in which neurons located close to the brain surface process low frequencies, and neurons located in ventral IC layers process high frequencies (Grinnell 1963; Friauf 1992; Jen and Chen 1998; Malmierca et al. 2008). There is one peculiarity in *C. perspicillata*’s IC: although ventral neurons are responsive to high frequencies they respond as well to low-frequency sounds (Beetz et al. 2017). In other words, neurons located in the ventral IC of this bat species are likely to display multi-peaked frequency-tuning curves and all neurons throughout the IC’s tonotopy respond (at least to some extent) to sounds whose carrier frequency lies in the 20–30 kHz range. It has been speculated that multi-peaked frequency tuning could allow neurons to respond to both, echolocation and communication sounds (Kanwal and Rauschecker 2007; Kössl et al. 2015). We reasoned that distress utterances produced by *C. perspicillata* could drive activity throughout the entire IC, since both dorsal and ventral neurons are responsive to frequencies ~ 20 kHz, corresponding to the peak frequencies of distress vocalizations (Hechavarría et al. 2016a).

Using laminar probes, we performed simultaneous recordings from dorsal and ventral IC areas in awake *C. perspicillata*. Our hypothesis was that, in response to echolocation sequences, the information provided by collicular neurons should be highest in ventral IC regions since echolocation sounds are mostly high frequency. On the other hand, information about communication sequences could be either highest in dorsal IC or equally distributed throughout the entire structure, due to the presence of multi-peaked frequency-tuning curves in ventral areas. The data revealed that ventral IC regions are more informative than dorsal regions not only about echolocation sounds, but also about communication, which was surprising given our original hypothesis. The ventral IC also contains the highest degree of response redundancy in pairs of neurons recorded simultaneously. This redundancy is tightly linked to signal correlations in the neurons recorded. Overall, the data presented in this article provide evidence for topographical representations of acoustic information and redundancy in the mammalian midbrain in naturalistic contexts.

## Results

The activity of 864 units (1 per penetration for each of the 16 recording sites on the silicon probe) was recorded from the central nucleus of the inferior colliculus (IC) in awake bats (species *C. perspicillata*) in response to pure tones and to 4 natural sound sequences (Fig. 1, for sequence parameters see Table 1). Note that since we did not perform anatomy in every animal, we cannot rule out the possibility that some units recorded do not belong to the central nucleus of the IC. Natural sequences consisted of three distress calls (*Seq1–3*) and one echolocation sequence. Distress calls are a type of communication sound used to advertise danger/discomfort to others (Russ et al. 1998, 2004; Eckenweber and Knörnschild 2016; Hechavarría et al. 2016a). The three distress sequences were chosen because they constitute typical examples of bats’ alarm utterances (Hechavarría et al. 2016a). Only one biosonar sequence was used, as echolocation is a stereotyped behavior that involves fixed action patterns as bats approach a target (Neuweiler 1990, 2003; Thies et al. 1998). The echolocation sequence was recorded in a pendulum paradigm in which a bat was swung towards a reflective wall (Beetz et al. 2016a). Distress and echolocation sequences have been used in previous studies characterizing the bat auditory system (Beetz et al. 2016a, 2017; Hechavarría et al. 2016b; Wohlgemuth and Moss 2016; Martin et al. 2017; García-Rosales et al. 2018; Macías et al. 2018). To restrict the study to units that responded to the calls, we considered only those units that carried at least 1 bit/s of information (Kayser et al. 2009) in response to at least one of the sequences studied (864 units out of 976). In addition, frequency-tuning curves were considered only for units that responded reliably to pure tones, i.e., they fired at least 6 spikes to all frequencies tested (814/864 units studied, ~ 94%). Manual inspection of the tuning curves was performed to double check that this arbitrary criterion provided consistent results.

**Table 1:** Basic temporal properties of the natural distress sequences used as stimuli

Seq	# of bouts	# of syllables	Sequence length [s]	Avg. length of bouts [ms]	Avg. length syllables [ms]	Avg. inter-syllable interval [ms]	Intensity (mean ± STD) [dB SPL]
1	1	9	0.51	510	69.2	141.4	90.2 ± 5.06
2	7	54	1.46	140	4.8	26.4	90.6 ± 2.45
3	8	122	1.96	176.6	2.9	15.7	90.2 ± 1.93
Echo	/	31	1.38	/	0.9	44.1	88.4 ± 5.0

The sound intensity was obtained considering each syllable individually per each sequence

## General properties of *C. perspicillata*’s auditory midbrain

**Fig. 2:**
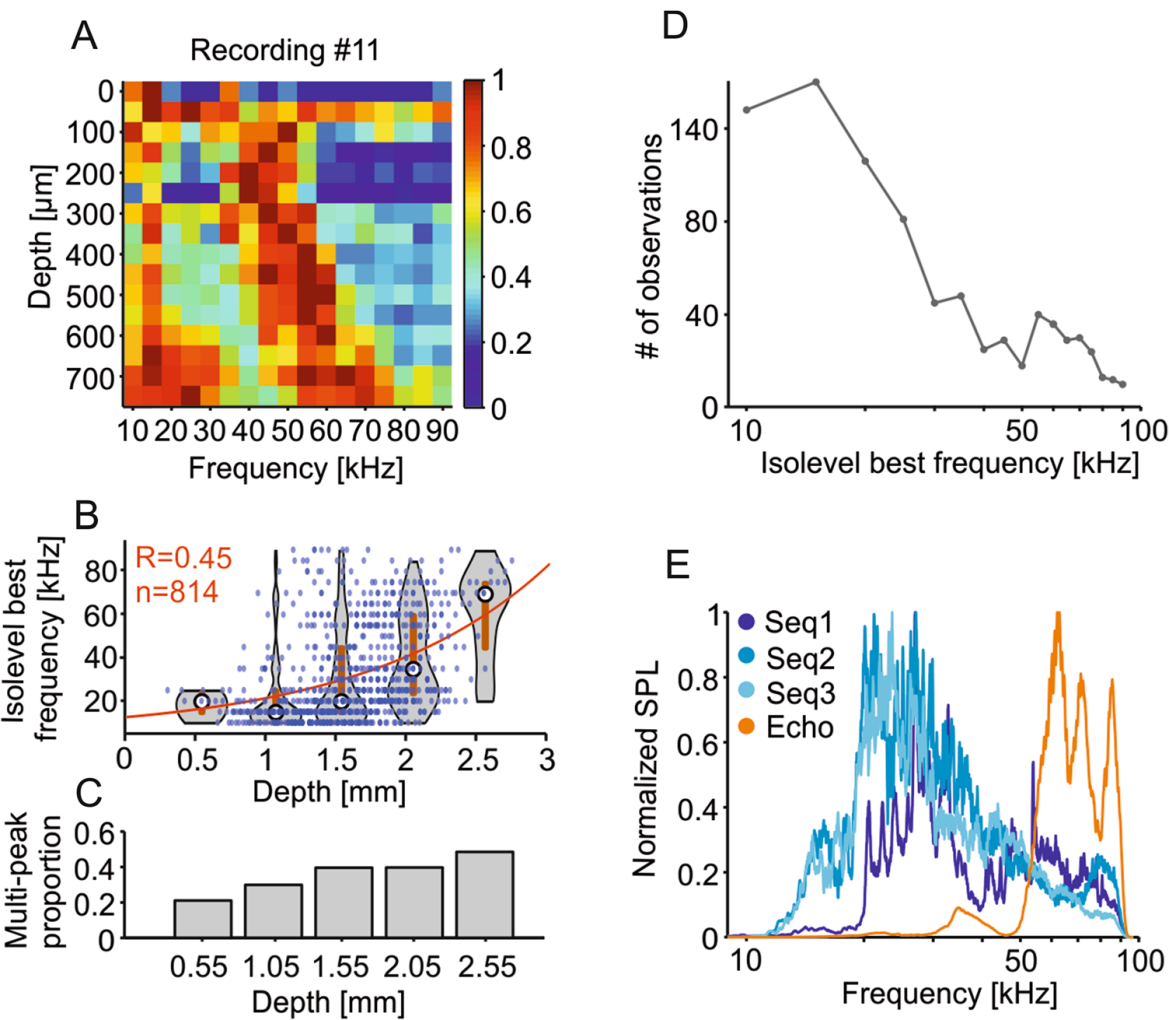
Tonotopy in the inferior colliculus. **a** Normalized (for each channel) number of spikes of the recording #11 plotted against the channel depth (relative to the most dorsal channel). **b** Scatter plot of the depth (in relation to the brain’s surface) and iso-level best frequency (iBF) of each unit. Note the general increase in iBF as the depth increases. In red, correlation coefficient (*R*) of the exponential curve fitted into the data with the bisquare robust method and equation: f(*x*) = 7.9*e*^0.0008x^ (*x*: depth in µm, *f(x)*: iBF in kHz). In the background are violin plots of the iBF per discretized depth (each violin considers units within a 0.5 depth range; note that the violin width is narrower than the considered depth range). The circle represents the median and the gray line represents the interquartile range. Only considered units that responded with > 6 spikes to all frequencies tested in the frequency-tuning protocol (*n* = 814). **c** Proportion of multi-peaked iso-level FTCs. Multi-peaked units had peaks in the frequency ranges of 10–35 kHz and 40–90 kHz (peak defined as > 85% of the maximum spike-count value). Note the increase of multi-peaked units in more ventral regions. **d** Histogram of the iBFs (*n* = 814). **e** Spectra of the natural calls used in this study with normalized SPL

Iso-level frequency-tuning curves (FTCs) were analyzed to confirm the tonotopy along the dorsolateral–ventromedial axis of the IC’s central nucleus (Grinnell 1963; Friauf 1992; Jen and Chen 1998; Malmierca et al. 2008; Beetz et al. 2017). The inferior colliculus is functionally organized in iso-frequency layers, with each layer being sensitive to a narrow range of frequencies, from low to high frequencies in the dorsolateral–ventromedial axis (Friauf 1992; Malmierca et al. 2008), which we approximate with dorso–ventral recordings. We analyzed the response to pure tones (frequencies from 10 to 90 kHz, steps of 5 kHz, 60-dB SPL) in terms of number of spikes to create iso-level FTCs. Figure 2a shows the results of an example recording and the iso-level FTCs obtained in all 16 channels simultaneously. Two peaks of high activity are evident, especially in deep IC areas. The high-frequency peak shifts to higher frequencies as the depth of the channels increases which demonstrates the tonotopy of the inferior colliculus (Fig. 2b, population data, 814 units, see exclusion criteria for frequency-tuning curves above). The low-frequency peak (10–30 kHz; Fig. 2a) occurs throughout all depths studied. Note that multi-peaked FTCs have been reported before in the bats’ IC (Casseday and Covey 1992; Mittmann and Wenstrup 1995; Holmstrom et al. 2007; Beetz et al. 2017), as well as in the AC of bats and other species (Sutter and Schreiner 1991; Fitzpatrick et al. 1993; Hagemann et al. 2011) and frontal auditory areas (López-Jury et al. 2019).

As expected from the known tonotopy of the IC, there was a positive correlation between neuronal best frequency (i.e., frequency that triggers the largest number of spikes; from now on, iso-level best frequency, iBF) and the depth of the channel that recorded the units (Fig. 2b). In addition, as IC depth increased, so did the probability of finding units with complex FTCs having more than one peak (multi-peaked FTCs, Fig. 2a, c). Note, however, that the two-peak population could be underrepresented, as above threshold levels could lead to inhibition at the characteristic frequency (Gaucher et al. 2020). At the population level, there was an overrepresentation of low iBF (10–30 kHz), likely influenced by the fact that neurons tuned to high frequencies were also responsive to low-frequency tones (Fig. 2d). Note that the range from 10 to 30 kHz corresponds to the peak frequencies in distress calls (Figs. 1 and 2e).

Based on the results obtained with pure tones, one could speculate that distress sounds (with peak frequencies at 20–30 kHz) should be best represented in dorsal IC layers or throughout the extent of the IC. On the other hand, echolocation sounds should drive strongest spiking in deep IC regions responsive to high frequencies.

## Ventral units in the inferior colliculus are better trackers of natural auditory streams

The main aim of this study was to assess whether there is a difference in information representation in response to natural sounds across IC depths. As stimuli, we used natural distress sequences, which carry most power in low frequencies (20–30 kHz), and an echolocation sequence with high power in frequencies ranging from 60 to 90 kHz (see stimuli in Fig. 1 and stimuli spectra in Fig. 2e).

**Fig. 3:**
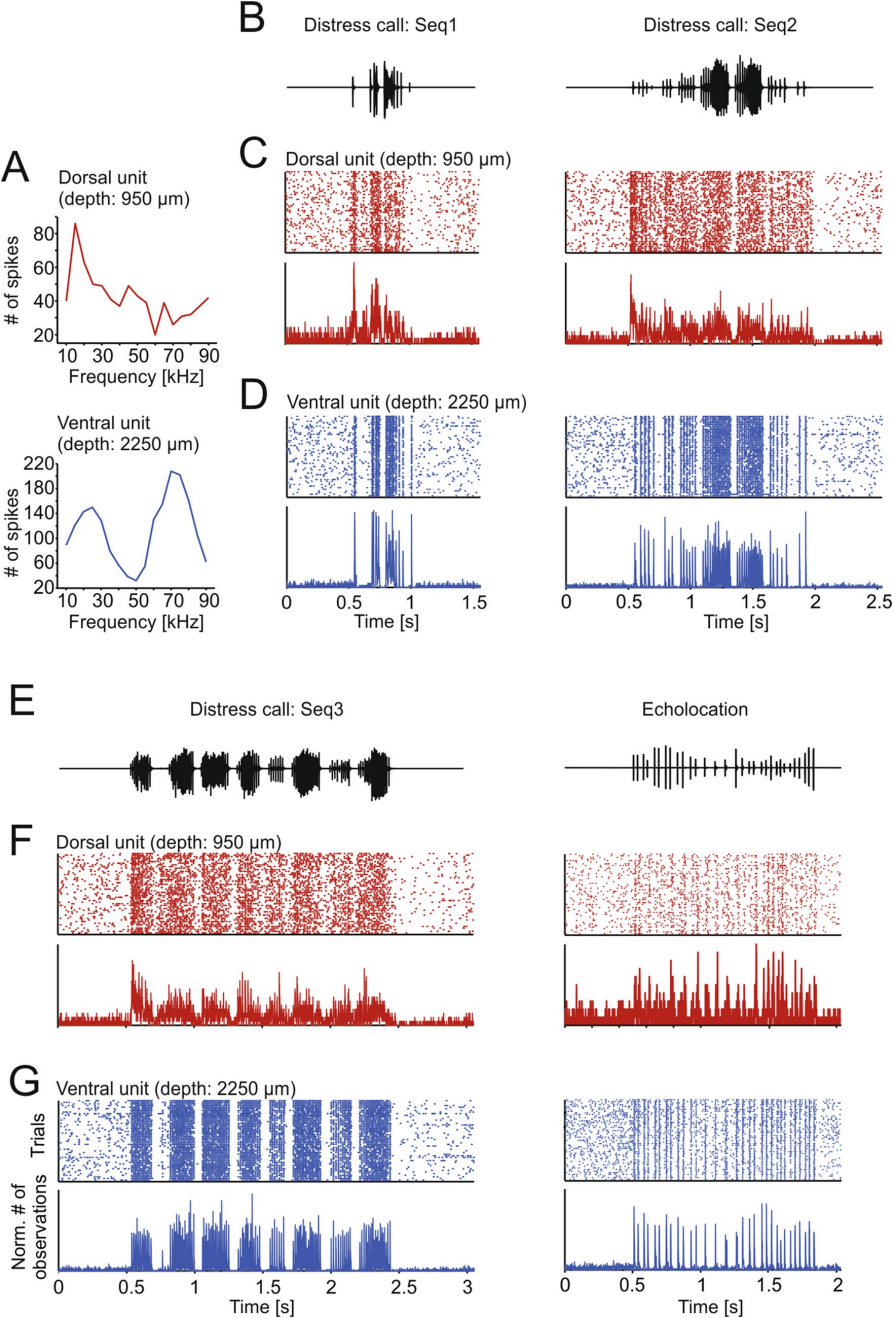
Ventral units represent more accurately the stimuli. **a** Frequency tuning curves of one exemplary dorsal unit (top, in red) and ventral unit (bottom, in blue). While the dorsal unit has a clear peak in the low frequencies, the ventral unit shows a double-peaked curve, one low- and the other high-frequency peak. **b**, **e** Oscillograms of the natural calls used as stimuli. **c**, **d**, **f**, **g** Raster plots (50 trials in total; top) and peristimulus time histogram (PSTH; 1-ms precision; bottom) of one dorsal unit (**c**, **f**; same as in **a** top) and one ventral unit (**d**, **g**; same as in **a** bottom) in response to the sequences shown in **b** and **e**. Note the higher precision and reliability in the ventral unit

A qualitative check of the neural responses to the sequences already revealed that dorsal units were worse in representing natural sound streams than ventral units, regardless of the type of stimulus presented (distress or echolocation). Ventral units appear more precise and reliable across trials in their responses to both distress and echolocation sequences (see example dorsal and ventral units in Fig. 3c, f and Fig. 3d, g, respectively).

**Fig. 4:**
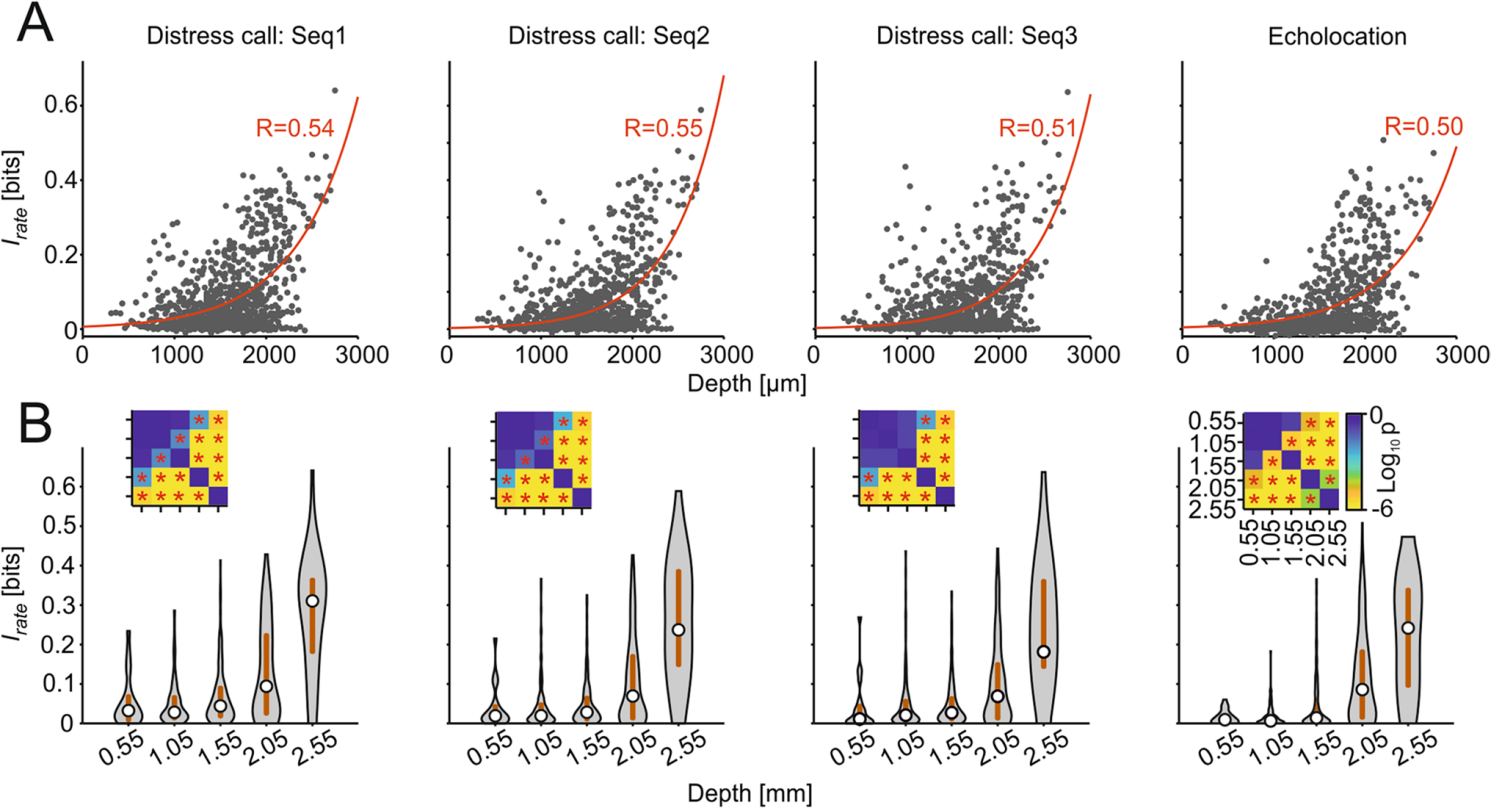
Ventral units carry the most information regardless of the stimuli. **a** Scatter plots of the information in the rate code plotted against the depth of the recorded unit for all sequences. In red is the exponential curve fitted to the data with the corresponding correlation coefficient value. **b** Violin plots of the information as in **a** with discretized depths for all sequences (median in circle, interquartile range in gray line). Each violin represents the information of the units comprised in 0.5-mm depth distance with center at the value stated in the labels. Statistical comparisons were performed by the FDR-corrected Wilcoxon rank-sum tests. The insets show p value matrices of all the statistical comparisons in a logarithmic scale. * *p*_corr_ < 0.05

Differences between dorsal and ventral units regarding the information they provide about natural sound streams were quantified by means of Shannon’s mutual information. Mutual information calculations capture all nonlinear dependencies between the response and the stimulus, and do not make assumptions about which stimulus features trigger the responses. The information based on a code defined by the spiking rate (*I*_rate_; see Methods) provided by the units increased exponentially with IC depth, regardless of the type of sequence (i.e., distress or echolocation) used as stimulus (Fig. 4). In other words, observing the neuronal response of ventral units reduces more the uncertainty about the stimulus than the response from dorsal units, and this trend was independent of the sounds heard. As shown in Fig. 4a, *I*_rate_ and IC depth had an exponential relation (the exponential fit had a higher adjusted r-squared value than the linear fit, adjusted *R*^2^_linear_ = 0.22 vs adjusted *R*^2^_exp_ = 0.25), with increasing *I*_rate_ with IC depth. Note that some units have negative information values due to the stringent effect of the bias correction on the information estimates (see Methods). To statistically compare the *I*_rate_ at different depths, we classified the *I*_rate_ estimates into five depth groups, and corroborated that the ventral units carry more information than dorsal units (Fig. 4b; FDR-corrected Wilcoxon rank-sum tests, *p* < 0.05) across all sound sequences tested. The same analyses with the additional segregation of single and multi-peaked units did not show substantial differences between these two groups (Fig. S2). Furthermore, we recalculated the information values considering the latency for each unit, i.e., shifting the response window so that it starts at the unit’s latency and found similar results to the ones obtained without latency correction (Fig. S3).

Finding an exponential relation between IC depth and mutual information was an unexpected result considering the tonotopic characteristics of the IC and the disparate spectral structure of the stimuli tested (echolocation vs. distress). Our results suggest the presence of a topographical representation of mutual information throughout the IC, presumably linked to the large complexity of receptive fields in ventral IC units and the complex spectra of natural sounds (see “Discussion”).

## Joint information in groups of neurons enables better representation of acoustic stimuli

The information provided by units recorded simultaneously was also quantified by means of joint information calculations (*I*_joint_, calculated in a total of 5237 pairs). *I*_joint_ measures the information considering responses in pairs of units as their combined activity, considering the identity of individual responses (i.e., which unit fired which spikes; see Methods). Note that a unit can be considered multiple times to form pairs with other units, since we recorded simultaneously from 16 IC loci.

**Fig. 5:**
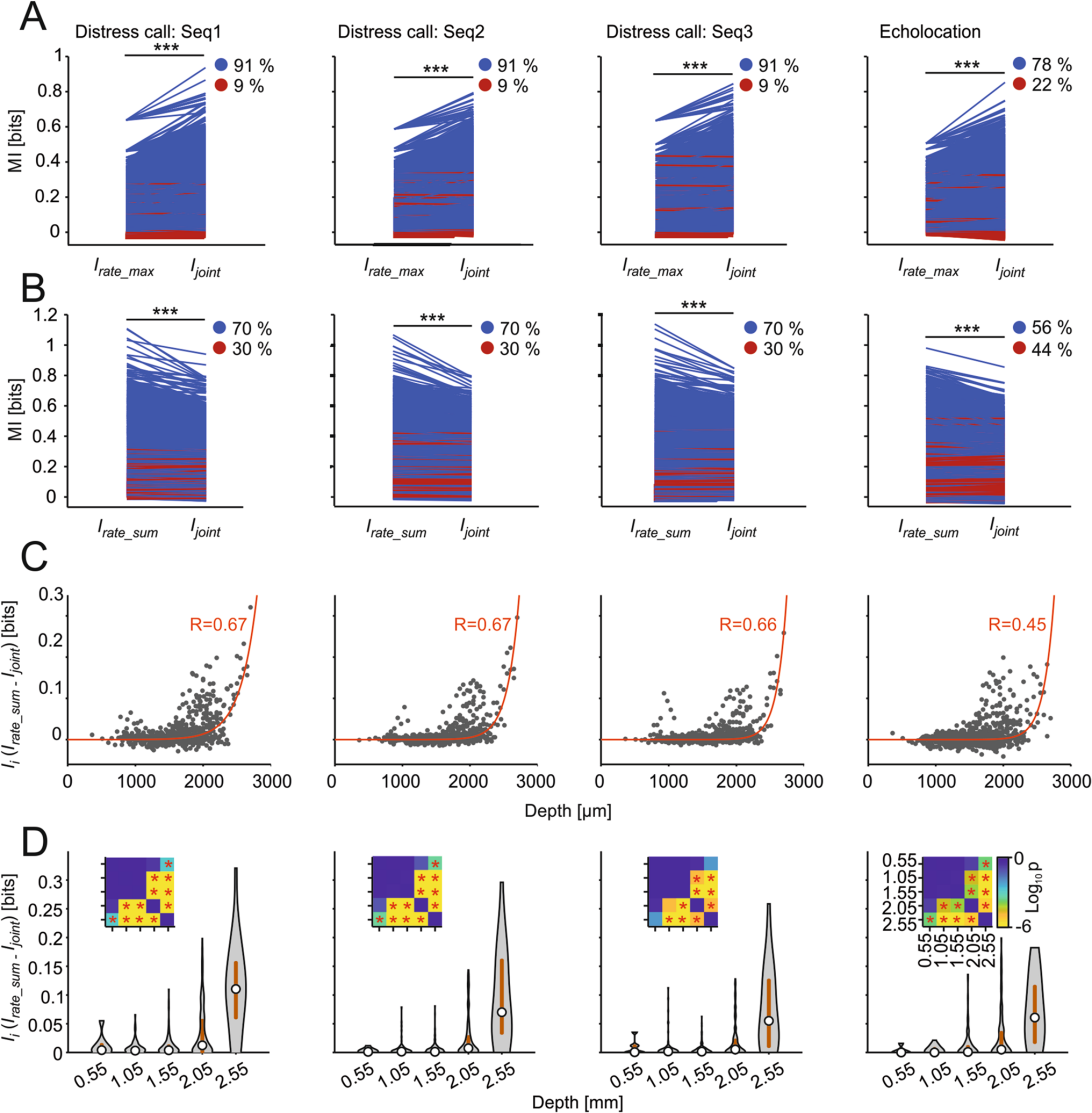
Redundancy increases with depth in simultaneously recorded units. **a** Quantitative analyses of the maximum *I*_rate_ of the units that form the pairs (*I*_rate_max_) and *I*_joint_ per sequence. Blue lines depict pairs which had *I*_rate_max_ < *I*_joint_ and red lines pairs which had *I*_rate_max_ > *I*_joint_. Percentage of pairs in each situation is shown on top. Statistical comparisons performed by FDR-corrected Wilcoxon signed-rank tests. ***p_corr_ < 0.001. **b** Quantitative analyses of the sum of information of the rate codes of each of the units that form the pairs (*I*_rate_sum_) and the *I*_joint_ per sequence. Blue lines depict pairs which had *I*_rate_sum_ > *I*_joint_ and red lines pairs which had *I*_rate_sum_ < *I*_joint_. Percentage of pairs in each situation is shown on top. Statistical comparisons performed by FDR-corrected Wilcoxon signed-rank tests. ***p_corr_ < 0.001. **c** Scatter plots of the *I*_i_ estimates (*I*_rate_sum_* – I*_joint_; *I*_rate_sum_ = *I*_rate(a)_ + *I*_rate(b)_) plotted against the intermediate depth of the pairs (only those pairs with units distanced by 100 µm were considered). In red is the exponential curve fitted to the data with the corresponding correlation coefficient value. **d** Violin plots of the *I*_*i*_ as in C with discretized depths for all sequences. Each violin represents the *I*_*i*_ of the pairs comprised in 0.5-mm depth distance with the center at the value stated in the labels. Statistical comparisons were performed by the FDR-corrected Wilcoxon rank-sum tests. The insets show *p* value matrices of all the statistical comparisons in a logarithmic scale. *p_corr_ < 0.05

The maximum *I*_rate_ estimates of the individual units that composed each pair (*I*_rate_max_) were compared to their *I*_joint_ (Fig. 5a). This comparison tests whether more information is provided by responses of pairs of units than by a unit separately. The results showed that *I*_joint_ was significantly higher than *I*_rate_max_ regardless of the stimulation sequence considered (FDR-corrected Wilcoxon signed-rank tests, *p* < 0.05). Thus, in the IC, the response of two simultaneously recorded units provides more information than the response of a single unit for the tested stimuli. Besides pairs, the information of the spike rate was also calculated for larger groups of units (Fig. S4): triplets (*n* = 16,703), quadruplets (*n* = 31,112) and quintuplets (*n* = 33,042). As expected, the information increased with the number of units considered (FDR-corrected Wilcoxon signed-rank tests, *p* < 0.05), i.e., with higher number of units used to calculate the mutual information, the more uncertainty of the stimulus was reduced.

## An information redundancy map exists in the auditory midbrain

To estimate if pairs of units carried redundant information, *I*_joint_ was statistically compared to the linear sum of *I*_rate_ of the units that formed the pairs (*I*_*rate_sum*_). *I*_*rate_sum*_ was significantly higher than *I*_joint_ in all the sequences considered (Fig. 5b; FDR-corrected Wilcoxon signed-rank tests, *p*_corr_ < 0.001), although to a lesser extent in the echolocation sequence, i.e., *I*_rate_sum_ was larger than *I*_joint_ only in 56% of the cases studied for echolocation vs. 70% for the three distress sequences. Overall, our results indicate that the population of simultaneously recorded units share information, at least to some degree. Thus, in response to natural sound streams, the auditory midbrain displays some degree of redundant information representation.

The degree of redundancy in unit pairs was quantified by computing the information interaction (*I*_i_) as the difference between the *I*_rate_sum_ and the *I*_joint_ for each neuronal pair. *I*_i_ calculations can yield three possible outcomes: (1) redundant representations (*I*_i_ > 0, indicating shared information between units); (2) synergy (*I*_i_ < 0, both units provide bonus information when studied simultaneously); and (3) independence (*I*_i_ = 0, units provide the same information when considered together and the sum when considered separately). Plotting *I*_i_ values vs. midbrain depth (mean depth of the two units forming each pair) revealed an exponential relation between these two variables, irrespectively of the stimulus presented to the bat (Fig. 5c). In other words, the highest *I*_i_ values (indicating more redundancy) are found in pairs of neurons recorded in deep-midbrain layers. This trend was statistically validated by comparing redundancy across depth groups (Fig. 5d; FDR-corrected Wilcoxon rank-sum tests, *p* < 0.05), which showed a significant increase of *I*_i_ from ~ 2-mm depth. Taken together, our results suggest that the ventral IC provides more informative, but also more redundant representations of natural incoming communication and echolocation sound streams.

## Redundancy is highest in nearby units and arises from signal correlations in unit pairs

**Fig. 6:**
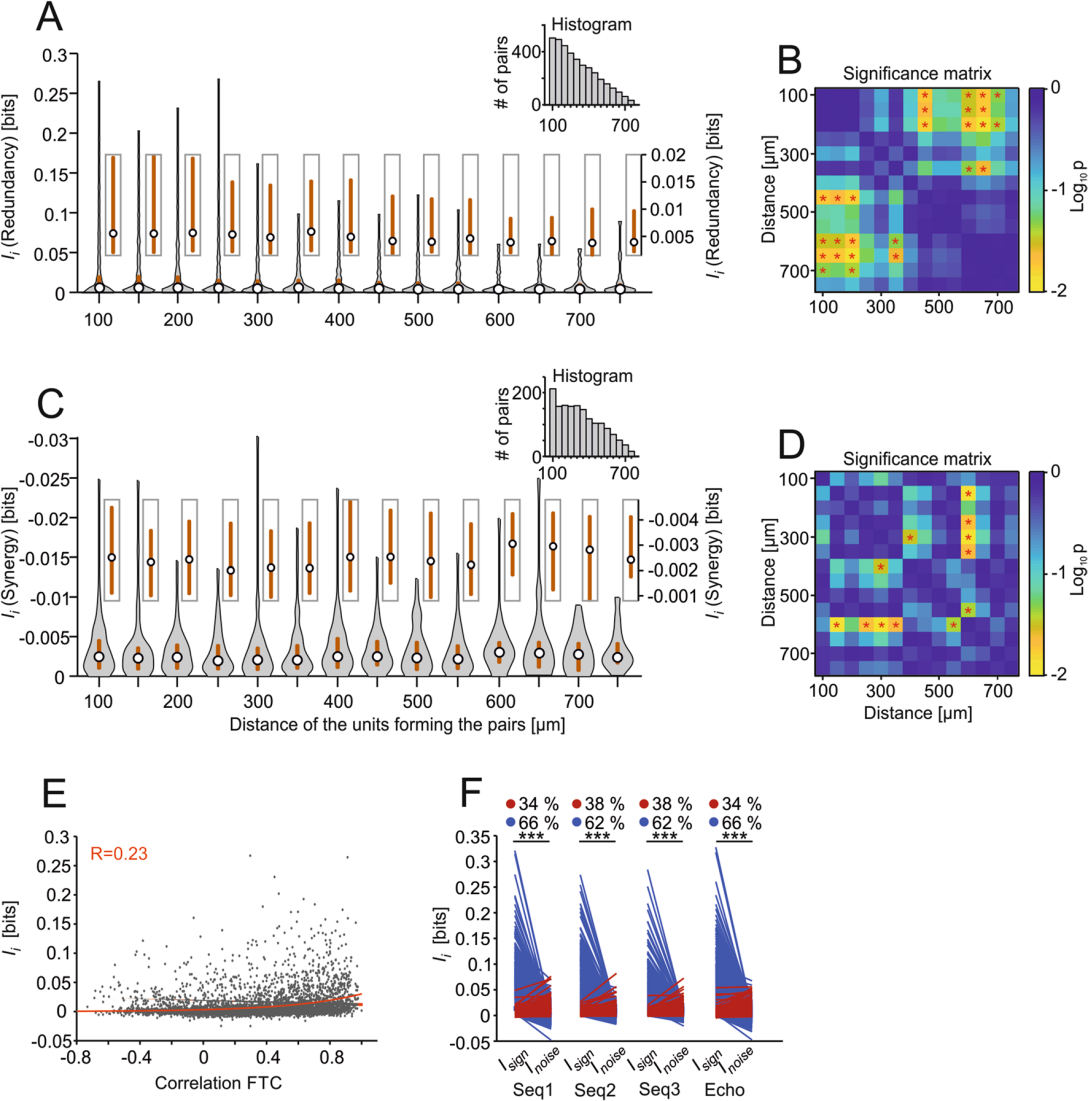
The redundancy is higher in nearby units and mostly comes from signal correlations. **a** Violin plot of the redundancy values (median in circle, interquartile range in gray line) for those redundant pairs (i.e., that had *I*_i_ > 0) displayed according to the distance between the units that form the pairs. Light boxes display a zoom-in of the median and interquartile range of the nearby violin plot (axis on the right). The inset shows the histogram of the pairs used for each distance. **b** *p* value matrix (FDR-corrected Wilcoxon rank-sum tests) with a logarithmic scale. *p_corr_ < 0.05 for statistical comparisons from (**a**). **c** and **d** with the same specifications for **a** and **b**, respectively, but for synergistic pairs (*I*_i_ < 0). **e** Scatter plot of the *I*_*i*_ estimates shown against the correlation coefficients between the frequency tuning of the units forming the pairs (*n* = 5237). In red is the exponential curve fitted to the data with the corresponding correlation coefficient value. **f** Quantitative analysis of the *I*_*i*_ broken down into signal (*I*_sign_) and noise (*I*_noise_) correlations for each pair and stimulus. Blue lines depict pairs which had *I*_sign_ > *I*_noise_ and red lines pairs which had *I*_sign_ < *I*_noise_. Percentage of pairs in each situation is shown on top. Statistical comparisons performed by FDR-corrected Wilcoxon signed-rank tests. FTC: frequency-tuning curve. *** *p*_corr_ < 0.001

To unveil the origins of the redundant representations observed, we separated unit pairs according to whether they showed redundant or synergistic interactions (*I*_i_ > 0 and *I*_i_ < 0, respectively). The *I*_i_ values were then analyzed considering the anatomical distance between the units forming the pairs (Fig. 6a–d). For this analysis, data from all stimulation sequences were pooled together. When considering only the redundant pairs, nearby units had higher redundancy levels than distant units (Fig. 6a). Even though the number of pairs decreased with inter-unit distance (see inset histogram), statistical comparisons between nearby and faraway units were significant (Fig. 6b). In the case of synergistic pairs (Fig. 6c, d), we did not observe a clear dependence of *I*_i_ with depth although there was a small increase in *I*_*i*_ values for pairs in which the units were located far away from each other. In other words, it appeared as if information redundancy was more likely to occur when units were close to each other, while synergy tended to be higher in pairs of distant units.

We also tested whether redundancy depended on units having similar iso-level frequency-tuning curves. To that end, *I*_i_ was analyzed considering the Pearson correlation coefficients between the FTCs of units forming the pairs (Fig. 6e). There was a moderate dependence between these two variables resulting in a correlation coefficient of 0.23 using an exponential fit (Fig. 6e). This shows a weak tendency towards higher correlated FTCs having more redundancy.

In a last step, we separated *I*_i_ into two components: (1) the contribution from signal correlations (*I*_sign_) and (2) from noise correlations (*I*_noise_). *I*_sign_ captures the similarity between the average response in the two neurons studied across different time frames of the same stimulation sequence, i.e., the degree to which the average response changes with the substimulus. *I*_sign_ is expected to be high in neurons with similar tuning properties (Averbeck et al. 2006). On the other hand, *I*_*noise*_ refers to the trial-by-trial variability in the responses (Averbeck et al. 2006). Noise correlations do not consider the impact of shared stimulation as they are quantified for “fixed” substimuli (Magri et al. 2009). While *I*_sign_ always results in redundancy, *I*_noise_ can lead to either redundancy or synergy. Our data show that in the IC, most of the redundancy between two units results from signal correlations (Fig. 6f). Thus, we can conclude that the redundancy observed in the IC is mostly stimulus-driven and does not necessarily represent an internal feature (noise) of the neuronal pairs.

## Discussion

In this study, we conducted simultaneous recordings of neuronal activity across the entire dorso–ventral extent of the inferior colliculus in awake bats presented with natural sound sequences. Our analysis focused on the spatial pattern of information representation at the midbrain level in response to natural sound streams. This is an important aspect for identifying which parts of the IC are instrumental for conveying information to other brain structures, such as the auditory thalamus, cortex and sensory-motor structures. Moreover, understanding how natural utterances are represented in the IC has direct translational implications, as this structure is a target area for prostheses aimed to help patients who cannot benefit from cochlear implants (Lim et al. 2009).

Our main findings are: (1) in bats, neurons carrying the most information about both distress and echolocation sequences are located ventrally in the IC; (2) unit pairs in ventral regions carry the highest redundancy as well; and (3) redundancy arises mostly from signal correlations in the units’ responses and is highest in nearby units with similar receptive fields.

## Ventral IC units have complex receptive fields and are highly informative about natural sound streams

In agreement with previous studies, we observed that the bat auditory midbrain contains units with multi-peaked FTCs (Casseday and Covey 1992; Mittmann and Wenstrup 1995; Holmstrom et al. 2007; Beetz et al. 2017). In *C. perspicillata*, these units fire strongly to both low-frequency (10–30 kHz) and high-frequency sounds with a response notch in between (see example tuning curves in Figs. 2a and 3a) and they are more likely to be found in ventral IC areas, as seen also in a study that used a multi-level frequency-tuning protocol (Beetz et al. 2017).

The fact that high-frequency units in the IC also respond to low frequencies has been described before in studies in other bat species such as the mustached bat, *Pteronotus parnellii* (Mittmann and Wenstrup 1995; Portfors and Wenstrup 1999; Macías et al. 2012). It appears that in some bat species, the tonotopic representation in the IC differs from the classical view so that, superimposed on the canonical dorso–ventral, low- to high-frequency axis, there is responsivity to low frequencies among the high-frequency region. In *P. parnellii*, multi-peaked frequency tuning is especially useful for integrating information about biosonar call and echoes in different frequency channels during target distance calculations (Suga and O’Neill 1979; O’Neill and Suga 1982; Mittmann and Wenstrup 1995; Portfors and Wenstrup 1999; Wenstrup et al. 1999; Macías et al. 2012). However, *C. perspicillata* (the species studied here) does not use multiple frequency channels for target distance calculations (Hagemann et al. 2010; Hechavarría et al. 2013; Kössl et al. 2014). Consequently, in this species, multi-peaked frequency tuning could offer advantages for representing communication sounds (i.e., distress) in widespread neuronal populations (Kanwal et al. 1994). Note that our data suggest that ventral IC neurons provide the highest information about distress and echolocation sequences. However, the latter does not necessarily imply that dorsal IC areas do not respond to some of the features in the natural sounds. Furthermore, delay-tuned neurons are located mostly in ventral IC regions (Wenstrup and Portfors 2011; Wenstrup et al. 2012). Whether delay tuning plays a role in representing communication sounds needs to be addressed in future studies.

According to our data, in the IC, neuronal information about natural acoustic sequences increase in an exponential continuum along the dorso–ventral axis. However, our study focused on the ability of neurons to represent information using a rate code. We did not quantify information using temporal codes. Future studies could clarify whether temporal and rate codes provide similar information patterns across IC depths. At least with the rate code, ventral IC units convey the most information about both echolocation and communication calls. This was an unexpected result due to the spectral differences between echolocation and communication calls and the tonotopic organization of the IC. Ventral IC neurons can provide informative responses about distress sounds because of several reasons. (1) A large proportion of ventral neurons respond to both low and high frequencies (i.e., multi-peaked frequency tuning), (2) the fact that distress calls carry energy at both low and high frequencies (albeit having their peak energy at ~ 22 kHz), and (3) a combination of both the former and latter. In other words, in multi-peaked units, the arrival of distress calls could activate inputs that correspond to both the low- and high-frequency peaks in the tuning curves. Note, however, that the amount of information provided by single- and double-peaked units in the ventral IC does not differ (Fig. S2), indicating that high information values is not solely correlated with the presence/absence of multi-peaked tuning.

Complex receptive fields could be beneficial for natural sound tracking because of several reasons. First, in response to distress, the simultaneous arrival of low- and high-frequency driven excitatory inputs would lead to spatiotemporal summation, which ultimately transduces into stronger responses (Magee 2000). Another possibility that could be considered is that in response to distress sounds, adaptation in the low- and high-frequency synapses occurs in an asynchronous manner. In the latter scenario, ventral IC neurons would always receive excitatory inputs since adaptation alternates between low- and high-frequency information channels. Note, however, that the data gathered using echolocation sequences do not differ much from that gathered using distress sequences (Fig. 4). Echolocation does not carry strong energy at frequencies below 45 kHz and the latter suggest that high-frequency inputs are sufficient for driving highly informative responses in the ventral IC. One could argue that echolocation requires specializations for precise temporal processing (Neuweiler 1990; Wenstrup and Portfors 2011; Kössl et al. 2014) and bats may profit from these adaptations even when listening to communication sounds. From the predictive coding framework (Remez et al. 1981; Bastos et al. 2012; Ayala et al. 2016), one could argue that echolocation processing implies a low-weighted prediction error which follows a “prior” hardwired in the system. In *C. perspicillata* such neural prior occurs in the form of a good sound tracking ability in ventral IC areas. Communication-sound processing would benefit also from this innate high informative prior.

Note that our data offer only insights into the final activity output of IC units but is not suited for assessing which of the above explanations (if any) contributes to the improved information representation in ventral IC layers. Information estimates used here only quantify the abilities of neurons to encode acoustic inputs, yet they do not capture the parameters of the stimulus the neurons are sensitive to (Borst and Theunisse 1999; Chechik et al. 2006; Timme and Lapish 2018). Thus, although both types of stimuli (distress and echolocation) showed similar patterns of information representation throughout the IC, different parameters of the two call types could contribute in different extents to the information maps observed.

## Possible origins of redundant information representation in the ventral IC

We observed that neurons in the ventral IC have complex receptive fields and carry high information content about natural sound sequences. However, the ventral IC also provides the most redundant information representations between units studied simultaneously. We show that redundancy in the ventral IC is linked to signal correlations, i.e., stimulus-induced activity correlations that arise when receptive fields overlap at least partially (Latham and Nirenber 2005; Averbeck et al. 2006). Common synaptic inputs can introduce both signal and noise correlations and could be the origin for the redundancy values reported here. In the cat’s IC, signal correlations have also been reckoned as the main contributor to the redundancy, which in turn decreases at higher stations of the auditory pathway (Chechik et al. 2006).

Our data indicate that in the IC, signal correlations are stronger than noise correlations, but this does not imply the absence of the latter. Signal correlations could relate to shared feedforward projections that dominate spiking during stimulus-driven activity and to both the crossed projections from the contralateral IC and local connections. On the other hand, noise correlations can arise from common input as well, in combination with stimulus-independent neuromodulation acting on each neuron individually (Belitski et al. 2010). Noise correlations could also reflect feedback projections, e.g., from the auditory cortex (Jen et al. 1998; Yan and Sug 1998) or the amygdala (Marsh et al. 2002), and they could regulate the IC’s processing at the single neuron level. In addition, the IC receives crossed projections from the contralateral IC and has a dense network of intrinsic connections (Malmierca et al. 1995) that could also influence the information interactions.

In the present study, we report high redundancy levels in pairs formed by close-by neurons (~ < 400 µm apart). This fact can be explained by the common inputs to neighbor neurons. Such common inputs might result from the IC’s tonotopy and they minimize wiring costs, an evolutionary adaptation linked to the formation of topographic maps (Chklovskii and Koulakov 2001, 2004). In the bat IC, there are also “synergistic neuronal pairs”, although consistent with the previous literature (Samonds et al. 2004; Narayanan et al. 2005), the predominant form of information interaction is redundancy. Studies have argued that the main advantage of redundant information regimes is that multiple copies of essentially the same information exist in the neural network activity patterns, i.e., similar information channels exist (Pitkow and Angelaki 2017). The latter gives room to the implementation of computationally different transformations on each information channel. Such transformations might be used by the bat auditory system to extract relevant stream features that go beyond the representation of the sounds’ envelope (e.g., occurrence of bouts in distress sequences or precise coding of echo-delays (Beetz et al. 2016a; García-Rosales et al. 2018)) in higher-order structures of the auditory hierarchy.

## Materials and methods

### Animals

For this study, four adult animals (three males, species *C. perspicillata*) were used. The animals were taken from the bat colony at the Institute for Cell Biology and Neuroscience at the Goethe University in Frankfurt am Main, Germany. The experiments comply with all current German laws on animal experimentation. All experimental protocols were approved by the Regierungspräsidium Darmstadt, permit #FU-1126*.*

### Surgical procedures

On the day of the surgery, the bats were caught at the colony and were anesthetized subcutaneously with a mixture of ketamine (10 mg/kg Ketavet, Pharmacia GmbH, Germany) and xylazine (38 mg/kg Rompun, Bayer Vital GmbH, Germany). Local anesthesia (ropivacaine hydrochloride, 2 mg/ml, Fresenius Kabi, Germany) was applied subcutaneously on the skin covering the skull. Under deep anesthesia, the skin in the dorsal part of the head was cut and removed, together with the muscle tissue that covers the dorsal and temporal regions of the skull. For fixation of the bat’s head during neurophysiology measurements, a custom-made metal rod (1-cm long, 0.1 cm diameter) was glued onto the skull using acrylic glue (Heraeus Kulzer GmbH), super glue (UHU) and dental cement (Paladur, Heraeus Kulzer GmbH, Germany). A craniotomy was performed 2–3 mm lateral from the midline above the lambdoid suture on the left hemisphere using a scalpel blade. The brain surface exposed was ~ 1 mm^2^.

During the surgery and the recordings, the custom-made bat holder was kept at 28º C with the aid of a heating pad. The surgery was performed on day 0, and the first recording was (at the earliest) on day 2. Further recordings were performed on non-consecutive days. On each experimental day, experiments did not last longer than 4 h, and during the recordings the animals received water every ~ 1.5 h. The animals participated in the experiments for a maximum of 14 days. After this time period, they were euthanized with an anesthetic overdose (0.1 ml pentobarbital, 160 mg/ml, Narcoren, Boehringer Ingelheim Vetmedica GmbH, Germany).

### Electrophysiological recordings

All recordings were performed in an electrically shielded and sound-proofed Faraday cage. Each recording consisted of three protocols: iso-level frequency tuning, spontaneous activity measurements and natural calls (three distress and one echolocation). In each recording day, the bat was placed on the holder and the rod on its skull was fixated to avoid head movements. Ropivacaine (2 mg/ml, Fresenius Kabi, Germany) was applied topically whenever wounds were handled.

On the first recording day, a small hole in the skull was made for the reference and ground electrodes on the right hemisphere in a non-auditory area. The same electrode was used for these two purposes by short-circuiting their connectors. The recording electrode (A16, NeuroNexus, Ann Arbor, MI), was an iridium laminar probe containing 16 channels arranged vertically with 50-µm inter-channel distance, 1.1–1.4 MΩ impendence, 15-µm thickness, and a 50-µm space between the tip and the first channel. The electrode was introduced 2–3 mm laterally from the scalp midline, ~ 1 mm caudal to the lambdoid suture (Coleman and Clerici 1987; Beetz et al. 2017), and perpendicularly to the surface of the brain, with the aid of a Piezo manipulator (PM-101, Science products GmbH, Hofheim, Germany). Before starting the recordings, the electrode was lowered down by 1.1–2.8 mm (depth measured from electrode’s tip to the brain’s surface). The tip’s depth was used as a reference to calculate the depth of all the channels. The position of the inferior colliculus was assessed by examining the responsivity to sounds across all recording channels. The sound used for testing for acoustic responsiveness was a short-broadband distress syllable covering frequencies between 10 and 80 kHz. The electrophysiological signals obtained were amplified (USB-ME16-FAI-System, Multi Channel Systems MCS GmbH, Germany) and stored in a computer using a sampling frequency of 25 kHz. The data were stored and monitored on-line in MC-Rack (version 4.6.2, Multi Channel Systems MCS GmbH, Germany).

### Acoustic stimulation

For the present study, three types of acoustic stimuli were used: pure tones, natural echolocation calls and distress calls from conspecifics. To assess the tonotopic arrangement of the IC, pure tones (10 ms duration, 0.5 ms rise/fall time) were presented at frequencies from 10 to 90 kHz in steps of 5 kHz at a fixed level of 60-dB SPL. The 17 pure-tone stimuli were played in a pseudo-random manner with a total of 20 repetitions per sound. Note that since the mammalian cochlear frequency-place map is logarithmic (Békésy 1960) and the pure tones used here are linearly spaced, the responses to low-frequency stimuli could have been undersampled.

The natural sounds comprised three distress and one biosonar sequences. The distress calls used as stimuli were recorded from conspecifics in the context of a previous study (see (Hechavarría et al. 2016a) for description of the procedures), and the echolocation call was recorded using a pendulum setup as in (Beetz et al. 2016b), which contains echoes at delays from 23 to 1 ms. These stimuli (also referred in this manuscript as *Seq1*, *Seq2*, *Seq3* and *Echo*) had durations of 1.51, 2.47, 2.93 and 1.38 s, respectively. Each stimulus was played 50 times in a pseudo-random order. The root-mean-square level of the syllables that formed the sequences spanned between 74.5- and 93.1-dB SPL (Table 1). The sequences were multiplied at the beginning and end by a linear fading window (10 ms length) to avoid acoustic artifacts. Sounds were played from a sound card (ADI-2-Pro, RME, Germany) at a sampling rate of 192 kHz, connected to a power amplifier (Rotel RA-12 Integrated Amplifier, Japan) and to a speaker (NeoX 1.0 True Ribbon Tweeter; Fountek Electronics, China) placed 30 cm away from the right ear. The speaker was calibrated using a microphone (¼-inch Microphone Brüel & Kjær, model 4135) recorded at 16 bit and 384 kHz of sampling frequency with a microphone amplifier (Nexus 2690, Brüel & Kjær). The resulting calibration curve is found in Fig. S1.

### Spike detection and sorting

Spikes were identified after filtering the data using a third-order Butterworth band-pass filter with cutoff frequencies of 300 Hz and 3 kHz. The threshold for spike detection was 6 MAD (median absolute deviation). Spikes were sorted using the open-source algorithm SpyKING CIRCUS (Yger et al. 2018), a method that relies on density-based clustering and template matching, and can assign spikes clusters to individual channels in electrode arrays without cluster overlap. For further analysis, the cluster with the largest number of spikes was used for each channel. This spike-sorting algorithm ensures that the same cluster is not considered in different channels. Spike-sorted responses are referred to as “units” throughout the manuscript.

### Information theoretic analyses

All the information theoretic analyses were performed using the Information Breakdown Toolbox (ibTB) (Magri et al. 2009). The capability of a neuron with a set of responses *R* to encode a set of stimuli *S* can be quantified using Shannon’s mutual information (*I*(*R*;*S*)) (Shannon 1948) using the following equation:

1 $$I\left( {R;S} \right) = \mathop \sum \limits_{s, r} P\left[ {r, s} \right]\log_{2} \frac{{P\left[ {r,s} \right]}}{P\left[ r \right]P\left[ s \right]},$$

where *P*[*s*] is the probability of presenting the stimulus *s*, *P*[*r*] is the probability of observing the spike count *r* and *P*[*r,s*] is the joint probability of presenting the stimulus *s* and observing the response *r*. The units of the mutual information are given in bits (when the base of the logarithm is 2). Each bit implies a reduction of the uncertainty about the stimulus by a factor of 2 by observing a single trial (Dayan and Abbot 2001). One variable provides information about another variable when knowledge of the first, on average, reduces the uncertainty in the second (Cover and Thomas 2006). Mutual information provides advantages in comparison to other methods as it is model independent and thus it is not necessary to hypothesize the type of interactions between the variables studied (Magri et al. 2009; Timme and Lapish 2018) and captures all nonlinear dependencies in any statistical order (Kayser et al. 2009).

The naturalistic stimuli presented here were chunked into non-overlapping time windows, the neuronal responses to which were used to estimate the information, as it has been similarly done and described in other studies (Steveninck et al. 1997; Belitski et al. 2008; Montemurro et al. 2008; Kayser et al. 2009; García-Rosales et al. 2018). The time window considered here for the substimuli (*T* = 4 ms) has been selected to make our calculations comparable to those from studies in the AC (Kayser et al. 2009; García-Rosales et al. 2018). To calculate the information contained in the firing rate of each unit (*I*_rate_), the number of spikes that occurred in response (*r*) to each substimulus (*s*_k_) was determined. The responses were binarized, i.e., they show if there is at least one spike (*1*) or none (*0*), *r* = *{0, 1}*. *P(r)* indicates the probability of firing (or not) and was estimated considering all the 50 trials of each sequence.

Information was quantified by two main neuronal codes: the rate code (*I*_rate_) and the information carried by the rate of two units (*I*_joint_) recorded simultaneously. The *I*_joint_ was calculated in the same manner as the *I*_rate_ with the difference that now the response (*r*) can take four forms (instead of two) as it keeps track of which neuron fired, therefore *r* = *{0–0, 0–1, 1–0, 1–1}*. As mentioned in the preceding text, the spike-clustering algorithm used in this paper considers the geometry of the laminar probe. Therefore, for each recording channel, a different spike waveform is allocated. To further make sure that a unit was not paired with itself during *I*_joint_ calculations, we considered only pairs of units from channels separated by at least a 100-μm distance. The mutual information was calculated as well for groups of three, four and five units recorded simultaneously.

To evaluate if the information carried by a unit pair was independent, redundant or synergistic, we calculated the information interaction (*I*_i_). *I*_i_ in pairs of neurons can be quantified by the sum of information conveyed by those neurons individually (*I*_rate_sum_ = *I*_rate(a)_ + *I*_rate(b)_) and the difference between information conveyed by the two neurons (*I*_joint_) (Brenner et al. 2000; Narayanan et al. 2005; Chechik et al. 2006):

2 $$I_{{\text{i}}} = I_{{{\text{rate\_sum}}}} - I_{{{\text{joint}}}}$$

If *I*_joint_ < *I*_rate_sum_ or simply if the *I*_i_ estimate is positive, the units carry redundant information; if *I*_joint_ = *I*_rate_sum_, the units carry independent information and if *I*_joint_ > *I*_rate_sum_, or if the *I*_i_ estimate is negative, they carry synergistic information. This compares the information available in the joint response to the information available in the individual responses.

The *I*_i_ was broken down into two components: the effects of signal and noise correlations. The signal similarity component (*I*_sign_) quantifies the amount of information specifically due to signal correlations, i.e., the degree to which the (trial-averaged) signal changes with the stimulus (Belitski et al. 2008). *I*_sign_ always reduces the *I*_i_ and typically arises when the neurons have similar tuning curves (Averbeck et al. 2006), i.e., when there are similarities between the responses of the units considered across substimuli (Magri et al. 2009). The noise correlation component (*I*_noise_) quantifies the impact of the trial-by-trial variability and can increase the *I*_i_, decrease it or leave it unchanged. Since it is measured at fixed substimulus, the *I*_noise_ disregards all effects attributable to shared stimulation (Magri et al. 2009):

3 $$I_{{\text{i}}} = I_{{{\text{sign}}}} + I_{{{\text{noise}}}} .$$

Information estimates were calculated by the “direct” method (Borst and Theunissen 1999), which requires a large amount of experimental data as it does not make any assumption about response probability distributions. As it is very difficult and improbable to observe all possible responses from the entire response set (*R*) (Panzeri and Treves 1996; Strong et al. 1998) due to the lack of unlimited number of trials, the quantities calculated with the estimated probabilities will always be biased. To account for that, the ibTB toolbox (Magri et al. 2009) uses the Quadratic Extrapolation (QE) procedure (Strong et al. 1998) and the subtraction of any remaining bias by a bootstrap procedure (Montemurro et al. 2008). In addition, for the *I*_joint_ tests, the Shuffling procedure (Panzeri et al. 2007) was also applied; which is also implemented in the ibTB toolbox.

To test the performance of the bias correction, simulated data with first-order statistics close to those of the real data were generated. For the *I*_rate_, spike responses were generated (inhomogeneous Poisson processes) with the same PSTH as each real unit used for the analysis (as in García-Rosales et al. 2018). Information was computed for the simulated data using the same parameters than for the original data, for all the neural codes used (*I*_rate_, and *I*_joint_ for pairs, triplets, quadruplets and quintuplets) and for different number of trials (4, 8, 16, 32, 50, 64, 128, 256, 512). According to our results of the performance of the bias correction on simulated data, the bias was negligible for the *I*_rate_, *I*_joint_ for pairs and triplets and had slightly negative for the *I*_joint_ for quadruplets and quintuplets, the information estimates underestimate the true information values for the last two variables.

### First-spike latency estimation

The first-spike latency was calculated as the time point in which the spiking rate in the observed response was significantly different from the expected spontaneous rate, assuming Poisson statistics (Chase and Young 2005), i.e., this method detects the first significant deviation from the expected spontaneous rate. For that, spikes from all *N* trials are collapsed (50 trials). The probability of observing a response of at least *n* spikes in a window *t*_*n*_ (after stimulus onset) assuming Poisson statistics is

4 $$P_{{{\text{t}}_{{\text{n}}} }} \left( { \ge n} \right) = 1 - \mathop \sum \limits_{m = 0}^{n - 1} \frac{{\left( {N\lambda t_{{\text{n}}} } \right)^{m} e^{{ - {\text{N}}\lambda t_{{\text{n}}} }} }}{m!},$$

where $\lambda$ is the spontaneous spike rate. Starting from the stimulus onset, the probability that each spike is the result of a stronger than chance rate deviation from the spontaneous rate (calculated during the 500 ms preceding the stimulus onset), is calculated as the probability that the spontaneous rate would have produced that spike as the last of *n* spikes in a window *t*_n_, where *n* ranges from 5 up to all the spikes considered in the particular window and *t*_n_ is the width of the window containing these spikes. The first time that these probabilities exceed a threshold of 10^–6^ is considered the unit’s latency to that particular stimulus.

The first-spike latency was used to shift the units’ responses, i.e., the onset of the responses was at the units’ latencies, for a recalculation of the *I*_rate_ (Fig. S3).

### Statistics

All statistical tests were performed using custom-written Matlab scripts (R2019b, MathWorks, Natick, MA). Non-parametric Wilcoxon rank-sum tests were used to assess the statistical difference of unpaired data and Wilcoxon signed-rank tests for paired data. Significant differences were considered when *p* < 0.05. Multiple comparisons were corrected for the false-discovery rate (FDR) using the Benjamini–Hochberg procedure (Benjamini and Hochberg 1995). In several figures, data are shown as violin plots (Hintze and Nelson 1998), which display distributions with density traces, median values with circles and interquartile ranges with thick lines. *r* (*r* = *z*/√*N*) was calculated as a non-parametric effect size measure (Fritz et al. 2012) to determine the importance of the effect in Fig. S3D.

## Supplementary Information

Below is the link to the electronic supplementary material.

Supplementary file1 (DOCX 17 KB)

Supplementary file2 (TIF 3134 KB)

Supplementary file3 (TIF 11794 KB)

Supplementary file4 (TIF 27356 KB)

Supplementary file5 (TIF 8027 KB)
