# Supplementary material for: Enhanced representation of natural sound sequences in the ventral auditory midbrain
Source: Brain Struct Funct. 2020 Dec 14;226(1):207–23. doi: 10.1007/s00429-020-02188-2 (PMC7817570; doi:10.1007/s00429-020-02188-2)
Supplement: Supplementary file 1 — Supplementary file1 (DOCX 17 KB) [file 429_2020_2188_MOESM1_ESM.docx]

**Supporting Information**

**Figure S1** **Calibration curve of the speaker used in the experiments**.

**Figure S2** **Information of single and multi-peaked units at different depths in the IC**. A) Violin plots of the information in the rate code for single (in orange) and multi-peaked (in blue) units in discretized depths for all sequences (median in circle, interquartile range in colored thick line). Each violin represents the information of the units comprised in 0.5 mm depth distance with center at the value stated in the labels. Considered units that responded with > 6 spikes for the frequency tuning protocol (n = 814). Multi-peaked units had peaks in the frequency ranges of 10-35 kHz and 40-90 kHz (peak defined as > 85% of the maximum spike-count value). B) *p*-value matrices of all statistical comparisons in a logarithmic scale. Statistical comparisons were performed by the FDR-corrected Wilcoxon rank-sum tests. * *p_corr_* < 0.05.

**Figure S3 Latency and mutual information calculated with consideration to the unit’s latency.** A) Histograms of the first-spike latencies for each stimulus tested (see Methods). B) Latency plotted against the depth for each unit and stimulus. C) Scatter plots of the information recalculated considering the latency of each unit (*I_rate-lat_*), i.e. the spike times consider as onset the latency of the unit instead of the stimulus onset. Note that the pattern of the information compared against the depth did not change after latency corrections. D) Quantitative analyses between the information as in the main text (*I_rate_*) and the one calculated with the corrected spike times (*I_rate-lat_*) Statistical comparisons performed by Wilcoxon signed-rank tests. Blue lines depict pairs which had *I_rate-lat_* > *I_rate_* and orange lines pairs which had *I_rate-lat_* < *I_rate_*. The effect size of each comparison is also shown (*r* = *z*/√*N*). Note that because of the large sample size (N = 864), reaching high levels of significance (small p-values) is not difficult, but the effect size of these comparisons is always either small or negligible.

**Figure S4 Mutual information for groups of units.** Violin plots of the mutual information carried by groups of units (pairs, triplets, quadruplets and quintuplets; median in circle, interquartile range in gray line) for each stimulus. Note that only units recorded from electrodes separated by distances > 100 µm were considered. Insets show the *P*-value matrix (FDR-corrected Wilcoxon rank-sum tests) with a logarithmic scale. * *p_corr_* < 0.001.
